# Nutrient and Hormonal Effects on Long Bone Growth in Healthy and Obese Children: A Literature Review

**DOI:** 10.3390/children11070817

**Published:** 2024-07-03

**Authors:** Sazid Hasan, Shahrukh Naseer, Mazen Zamzam, Hashem Mohilldean, Colin Van Wagoner, Ahmad Hasan, Ehab S. Saleh, Virginia Uhley, Suzan Kamel-ElSayed

**Affiliations:** 1 School of Medicine, Oakland University William Beaumont, Rochester, MI 48309, USA; 2 Department of Orthopedic Surgery, Detroit Medical Center, Detroit, MI 48201, USA; 3 Department of Orthopedic Surgery, Beaumont Hospital, Royal Oak, MI 48073, USA

**Keywords:** pediatrics, obesity, bone growth, nutrition

## Abstract

Longitudinal bone growth is mediated through several mechanisms including macro- and micronutrients, and endocrine and paracrine hormones. These mechanisms can be affected by childhood obesity as excess adiposity may affect signaling pathways, place undue stress on the body, and affect normal physiology. This review describes the physiology of the epiphyseal growth plate, its regulation under healthy weight and obesity parameters, and bone pathology following obesity. A literature review was performed utilizing PubMed, PMC, NIH, and the Cochrane Database of Systematic Reviews pertinent to hormonal and nutritional effects on bone development, child obesity, and pathologic bone development related to weight. The review indicates a complex network of nutrients, hormones, and multi-system interactions mediates long bone growth. As growth of long bones occurs during childhood and the pubertal growth spurt, pediatric bones require adequate levels of minerals, vitamins, amino acids, and a base caloric supply for energy. Recommendations should focus on a nutrient-dense dietary approach rather than restrictive caloric diets to maintain optimal health. In conclusion, childhood obesity has profound multifaceted effects on the developing musculoskeletal system, ultimately causing poor nutritional status during development. Weight loss, under medical supervision, with proper nutritional guidelines, can help counteract the ill effects of childhood obesity.

## 1. Introduction

During childhood, endochondral ossification within the epiphyseal growth plate results in long bone formation. Long bones, such as the femur and humerus, grow throughout puberty until the maturation of the growth plate. This growth and maturation are the result of several mediators including macronutrients, micronutrients, as well as endocrine and paracrine hormone signaling [1].

This complex balance of nutrients and hormones and their downstream effects on the development of long bones can be affected by obesity. Through effects on cellular signaling mechanisms, overall nutritional status in the growing skeleton, and undue stress placed on the body, obesity can lead to problems in growing bone [2]. Currently, 17% of children in the US present with excess adiposity. This places children at risk for underdeveloped bones, stunted growth, and many pathologic musculoskeletal abnormalities.

The objectives of this literature review were to describe the normal physiology of the epiphyseal growth plate, discuss factors influencing growth plate physiology and bone mineral density, along with how such factors change under healthy weight and obesity parameters, and discuss bone pathology following obesity. Furthermore, we hope to elucidate recommendations on treating childhood obesity.

## 2. Materials and Methods

A search for the most relevant literature was performed using the following key words: nutrition, obesity, pediatrics, surgery, long bone growth, spondylitis, discitis, spondylodiscitis, osteomyelitis, and epidural abscess. The search for the relevant literature was performed using these search engines: PubMed, ScienceDirect, Ovid, Springer, and Google Scholar. The search included the relevant studies published between 1990 and 2023.

## 3. Results

### 3.1. The Normal Structure and Function of the Epiphyseal Growth Plate

At the ends of long bones, such as the femur or the humerus, lies the growth plate, or the physis. This cartilaginous area of growth allows for endochondral ossification of the skeleton—the process of systematically replacing cartilage with growing bone. This provides the foundation for setting the length of the mature bone, as well maintaining bone strength, flexibility, and bone homeostasis [3].

**Figure 1:**
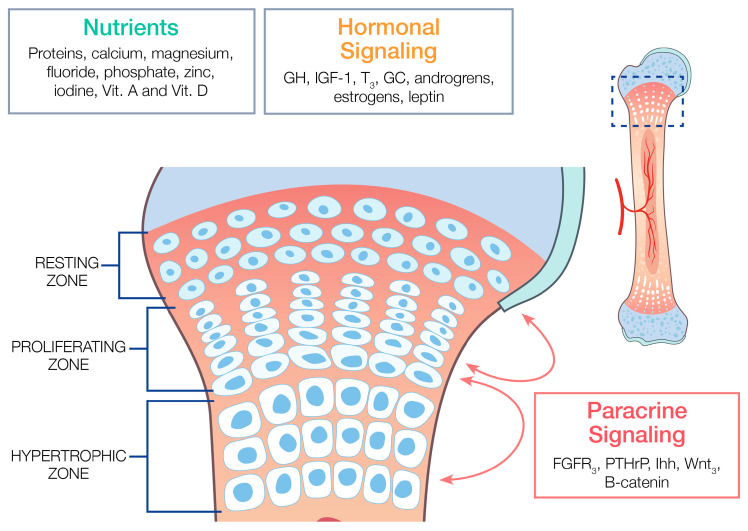
The epiphyseal growth plate. The diagram shows the epiphyseal growth plate structure in children. It is divided into three major zones: the resting zone, the proliferative zone, and the hypertrophic (maturation) zone. The growth plate is regulated by a complex network of nutrients, endocrine hormones, and local paracrine factors. GH: Growth hormone. IGF-1: Insulin-like growth factor-1. T_3:_ Triiodothyronine. FGFR_3_: Fibroblast growth factor receptor 3. PTHrP: Parathyroid hormone-related peptide. Ihh: Indian hedgehog. Wnt_3_: Proto-oncogene protein Wnt-3.

The physis is a hyaline cartilage plate that is divided into three major zones: the resting zone, the proliferative zone, and the hypertrophic (maturation) zone (Figure 1). The resting zone is found closest to the epiphysis and consists of non-dividing cells with the weakest blood supply of the three zones. It serves as a source for progenitor cells responsible for matrix production in the form of Type II collagen, and can generate new, rapidly dividing chondrocytes [4].

Below the resting zone is the proliferative zone, which is composed of highly metabolic proliferative cells arranged into columns. This zone has the most extensive blood supply, allowing for both nutrients and hormones to reach their targets. In newborns there is rapid cellular division, to the rate of 50 linear cm/year, slowing to around 5 linear cm/year by the age of 10. Following the pubertal growth spurts, the growth plate closes and linear growth of long bones is complete [5,6].

Finally, the hypertrophic (maturation) zone lies closest to the calcified bone of the metaphysis. The chondrocytes found here are hypertrophic and highly metabolically active, producing collagen X. These cells express high levels of vascular endothelial growth factor to aid the skeleton in initiating vascular invasion. These chondrocytes eventually undergo apoptosis, and the vascular invasion culminates with osteoblasts replacing the calcified cartilage with bone (calcification) [7,8]. This primitive, unorganized bone is called woven bone, which is replaced with highly structured, organized bone known as lamellar bone. The process of bone remodeling takes place constantly, and many metabolic processes are involved in maintaining homeostasis between bone growth and bone destruction [9].

### 3.2. The Nutritional and Hormonal Regulation of the Epiphyseal Growth Plate under Normal Weight and Obesity Parameters

#### 3.2.1. Major Macronutrients and Micronutrients

Macro- and micronutrients are essential to support the proper growth and development of bones. A quality diet ensuring an adequate supply of these nutrients has been linked to stronger bones and height [10]. Aside from the high metabolic activity required for the formation of bones throughout early childhood and puberty, a lack of proper amino acids, vitamins, and minerals can cause stunted growth [10]. The current Recommended Dietary Allowances (RDAs) for optimal growth are reported in Table 1, and vary based on age, sex, estimated energy expenditure, and health conditions [11].

Nutrient-dense dietary approaches for children should focus on evidence-based guidelines such as those of the US Department of Health and Human Services, as well as the US Department of Agriculture’s Dietary Guidelines for Americans (DGAs), and the Institute of Medicine’s Dietary Reference Intakes (DRIs) [12,13]. Aside from a lack of proper nutrients, overall caloric requirements vary based on age and should be properly monitored to ensure optimal bone growth. Restrictive caloric diets, as well as over-consuming daily recommended caloric intakes can cause altered metabolic function and bodily growth [1,11].

The current DRIs for the estimated energy requirements and RDAs for protein requirements to support growth, bone development, and health for infants and children are found in Table 1 based on relevant studies [11,12,13].

Even with adequate caloric intake, deficiencies in protein have been shown to impair bone growth [14]. IGF-1 has been shown to reduce in function with acute protein deficiency and in protein energy malnutrition in children [15,16,17]. Studies in children with protein-calorie malnutrition have demonstrated elevated GH levels and low IGF-1 levels which are normalized after adequate protein intake is met [18,19]. Essential amino acids have been shown to also act as signaling factors in regulatory patterns associated with growth [20]. The amino acids Arginine and Lysine are known to stimulate GH and IGF-1; however, studies on intake and linear growth are currently lacking [21].

##### Minerals

A proper dietary intake of minerals is essential for the development of long bones. In Table 2, the most important mineral factors for the development of bones are listed and sorted by age requirements based on pertinent studies [13,22,23].

- Calcium

The dietary requirements for calcium vary throughout the life cycle and are based on the skeleton’s needs for development and maintenance [12]. Calcium is a major mineral component of bone, providing strength and structure. The regulation of calcium is based on dietary intake, vitamin D signaling, and PTH signaling [24,25]. Calcium deficiency leads to a disorganized structure and promotes focal breaks within the growth plate [22].

Several studies have found that the average American diet is lacking in adequate calcium levels. Furthermore, there is a marked relationship between obesity and calcium levels, wherein individuals with low body fat percentages eat a higher RDA of calcium compared to the diet of an obese individual. Furthermore, lipogenesis due to elevated levels of PTH can lead to a cycle of weight gain and calcium depletion from bones. Lastly, there has been recorded reciprocal messaging on elevated dietary calcium on limiting fat absorption [26].

- Magnesium

Magnesium is a critical mineral involved in regulating bone development as a component of the bone mineral matrix and in regulating hormonal status [27,28]. With depleted magnesium levels, there is a decrease in osteoblastic activity, decreased bone formation, a thinning of the epiphyseal plate, and increased fragility.

Magnesium is also a required component for parathyroid hormone release, which is an endocrine regulator of calcium and phosphate levels. A deficiency in magnesium will reduce parathyroid levels, which in turn lowers bone mineralization in all age groups [22,27].

Studies have shown that obese patients are more likely to be deficient in magnesium, likely due to poor dietary intake of essential minerals. Conversely, magnesium supplementation prevents the accumulation of adipose tissues. With magnesium loss, glucose oxidation is impaired and oxidative metabolism is hindered, weakening the availability of energy stores for bone formation. Magnesium is needed for vitamin D optimization [29]. With magnesium deficiency, the growth plate has been found to lose up to 33% of the width, and chondrocyte columns decrease in number and length, pointing towards defective bone growth [30].

- Fluoride

Fluoride has been shown to stimulate bone formation through direct effects on osteoblasts [31]. It can accumulate in the developing skeleton of children at a faster rate than in adults [32]. It has also been found to increase the number and diameter of chondrocytes found within the growth plate [33]. Furthermore, fluoride promotes HSPG and FGFR2 in the growth plate and inhibits the Ihh/PTHrP signaling loop during endochondral ossification [34].

Currently, there are no studies showing the effects of obesity on fluoride levels; however, there has been a connection between increased fluoride intake and obesity in children [35].

- Phosphate

Phosphate has been shown to have a significant impact on bone development and maintenance because of the role it plays in the process of apoptosis of mature chondrocytes in the growth plate [32,36]. An excess of phosphate levels can increase the signaling of PTH, causing resorption of bone, while a relative deficiency may lead to the development of rickets [37]. Hypophosphatemia prevents apoptosis in the hypertrophic cells in the growth plate, which results in the accumulation of hypertrophic cells in the growth plate and forms rachitic bone. Phosphopenic rickets can also develop in children and adolescents, which can be caused by renal phosphate wasting [38]. Phosphate has been found to have an inverse association with BMI and overall body fat percentage, with a stronger relationship in women, creating a link between obesity and the formation of rickets [39].

- Zinc

Zinc has been shown to affect bone metabolism and to have direct effects on IGF-I and GH [40,41,42]. Through these signaling mechanisms, zinc plays an important role in promoting the bone growth rate (including weight and length), growth plate diameter and thickness, and cell count in the hypertrophic (maturation) zone [43,44]. These metrics, and their counter-effects in a state of zinc deficiency, further highlight the importance of zinc in the GH pathway.

In obese patients, zinc is yet another micronutrient found in inadequate quantities. Both plasma levels and dietary intake are markedly decreased, highlighting the need for proper nutrient-based guidelines in ensuring healthy bone development in children [45]. A zinc deficiency will also increase leptin production, increasing the inflammatory changes found with excess adiposity [46].

- Iodine

Iodine is an important factor of thyroid hormones; thus, the effects mediated relate to how iodine levels affect thyroid function. Thyroid hormones can affect bone calcium metabolism either by a direct action on osteoclasts or by acting on osteoblasts [23]. They also promote the differentiation and maturation of chondrocytes and osteoblasts. Iodine deficiency leads to decreases in thyroid hormone production and linear bone growth [47,48].

There has been limited research indicating that increased adiposity and the resultant lymphocyte infiltration of the thyroid parenchyma can weaken the function of the thyroid. Further research has been inconclusive on whether obesity results in greater or lesser urinary excretion of iodine, so it cannot be determined if iodine levels are directly correlated with obesity or if there are greater socioeconomic and dietary factors at play [49].

##### Vitamins

A proper dietary intake of vitamins is also essential for the development of long bones. In Table 3, the most important vitamins for the development of bones are listed and sorted by age requirements based on pertinent studies [13].

- Vitamin A

Retinol, retinal, and retinyl esters are important for bone development and influence both osteoblast and osteoclast activities. Vitamin A metabolites can induce the differentiation of osteoblastic progenitor cells and stimulate the mineralization of bone, promoting osteogenesis [50]. Of note, excess vitamin A activity can cause premature fusion of the growth plate due to negative regulation of osteoblastic activity, so care must be taken to ensure vitamin A is consumed in adequate and not excessive quantities [51]. Deleterious effects may include excessive bone resorption, thinning, and fragility [50].

Obese individuals have been found to have lower vitamin A levels than normal-weight individuals. This deficiency can increase the expression of leptin and further promote adiposity and inflammation [52]. Consequently, there are negative downstream effects on proper bone formation in obese patients.

- Vitamin C

Vitamin C affects trabecular bone formation by regulating gene expression in osteoblasts. Vitamin C also stimulates the production of the extracellular matrix, alkaline phosphatase, osteocalcin, and RANKL [53]. According to one study, vitamin C use prevents the harmful effects of glucocorticoids on the growth plate of animal models [54]. Vitamin C deficiency can lead to scurvy, which is characterized by multiple complications, including impaired bone growth and bone pain [55].

There is an inverse relationship between BMI and plasma vitamin C. Lower vitamin C consumption and status is also linked to higher dietary fat and sugar levels. Another explanation may be that systemic inflammation results in higher oxidative stress, increasing the body’s utility of vitamin C stores [56].

- Vitamin D

Vitamin D plays an important role in the homeostasis of calcium in the body. It enhances calcium absorption in the small intestine via paracellular and transcellular pathways. Vitamin D-dependent calcium transport proteins such as TRPV6 and calbindin 9k are found on surfaces in enterocytes and are heavily regulated by vitamin D levels [57].

The physiological role of vitamin D on the growth plate remains unclear. However, vitamin D is required for normal bone formation and normal mineralization [55]. Obese children were found to display vitamin D deficiency, which if prolonged can lead to rickets [55]. In vitamin D deficiency, the length, morphology, and mineral content of the growth plates and long bones are impaired, with widening and disorganization of the hypertrophic zone. There is also a decrease in hypertrophic chondrocyte apoptosis due to low serum phosphate levels secondary to vitamin D deficiency [58]. In addition, the removal of hypertrophic chondrocytes is reduced because of defective osteoclasts and decreased vascular invasion, leading to an overall enlargement of the cartilaginous epiphyseal growth plate and altered growth plate remodeling [55,58]. This deficiency will clinically lead to rickets (soft weak bone), which in turn can cause skeletal deformities such as bowed legs, knock knees, thickened wrists and ankles, soft skull bones, scoliosis, and pectus carinatum.

#### 3.2.2. Major Endocrine Hormones

- Growth Hormone and IGF-1

Growth hormone is a peptide hormone produced by somatotroph cells in the anterior pituitary gland. It is secreted in a pulsatile manner and is stimulated via GH-releasing hormone (GHrH) from the hypothalamus, while inhibited by GH-inhibiting hormone (Somatostatin).

In humans, the GH receptor (GHR) is expressed in large amounts in the liver, adipose tissue, heart, kidneys, intestine, lung, pancreas, cartilage, and skeletal muscle, where it induces the synthesis of IGF-1 [59]. Most circulating IGF-1 is synthesized by the liver; however, GH acts on the resting zone directly to recruit proliferating chondrocytes and to stimulate local production of IGF-1. Several studies indicate that locally acting GH, glucocorticoids (GCs), and T_3_ regulate the expression of IGF-1 and its receptor in the physis directly [60,61,62].

This IGF-1/physis interaction is a key regulator for endochondral ossification, promotes clonal expansion of proliferative chondrocytes via an autocrine/paracrine manner, and enhances the synthesis of proteoglycan synthesis [60,61].

With obesity, the spontaneous and stimulated GH secretion is decreased, but IGF-1 levels are increased, allowing for normal growth in obese children. With the achievement of normal body weight, the abnormality of GH secretion is completely reversed [63].

- Thyroid Hormones

T_3_ is a tyrosine derivative (amine) hormone produced by the follicular cells of the thyroid gland. Most circulating T_3_ is derived via the metabolism of T_4_ by thyroid deiodinases. The level of circulating thyroid hormone is regulated by the hypothalamic-pituitary–thyroid (HPT) axis. Its secretion is controlled by a negative feedback mechanism of thyrotropin-releasing hormone (TRH) in the hypothalamus, and thyroid stimulating hormone (TSH, thyrotropin) in the anterior pituitary.

Thyroid hormone nuclear receptors are expressed in almost all tissues, though the different relative concentrations vary at different stages of development and with age [64]. As the epiphyseal growth plate is directly responsive to T_3_, thyroid hormones are essential for longitudinal bone growth. Thyroid hormones bind to their receptors in resting and proliferating chondrocytes to recruit chondrocyte progenitor cells, inhibit chondrocyte proliferation, stimulate chondrocyte differentiation, increase collagen X production, and enhance cartilage matrix mineralization [65,66,67].

In obese children, the pituitary thyroid axis is altered, resulting in moderately increased levels of TSH, T3, and T4 [68,69]. These changes in thyroid hormone secretion can cause delayed physeal closure, predisposing obese children to malformed bones and pathology such as slipped capital femoral epiphysis [70].

- Glucocorticoids (GCs)

Glucocorticoids are steroid hormones produced by the adrenal cortex. Their secretion is regulated by corticotropin-releasing hormone (CRH) from the hypothalamus and adrenocorticotropic hormone (ACTH) from the anterior pituitary. Physiologically, GCs facilitate normal bone growth directly by binding their receptors in the physis, and indirectly through their mediations of the GH-IGF-1 axis.

They are widely used as anti-inflammatory and immunosuppressive drugs in children, and excess levels have been known to have growth-reducing effects on the growth plate. This is via inhibition of chondrocyte proliferation, prolonging the resting phase, and stimulation of chondrocyte apoptosis [61,62,71,72].

Obese children were found to display long-term activation of the hypothalamus–pituitary–adrenal (HPA) axis, causing a higher level of cortisol secretion [73,74]. This excess causes reduction in GH secretion, inhibition of proper bone formation, and stimulation of bone resorption [73]. In addition, excess glucocorticoid levels negatively affect calcium metabolism [75].

- Androgens and Estrogens

Androgens are secreted from the testicular Leydig cells in males and from the adrenal glands in both males and females. They are steroid hormones that stimulate longitudinal bone growth and play a role in the pubertal growth spurt. The direct effect of androgens is via binding to their receptors which are expressed in all layers of the growth plate in both males and females [76]. Indirectly, adrenal androgens are aromatized to estrogens in tissues containing such aromatase enzymes, including adipose and the growth plate [77].

Gonadal secretion of estrogen is stimulated by the pulsatile secretion of gonadotropins (LH and FSH) from the anterior pituitary. This pulsatility is accentuated with the onset of puberty. Recent studies have hypothesized that estrogen at low concentrations stimulates skeletal muscle growth and maturation in both males and females. Furthermore, this low concentration stimulates GH secretion, chondrocyte clonal expansion, and proliferation of chondrocytes. Continuous exposure to higher levels of estrogen will cause the closure of the growth plate, osteoblast invasion of the growth plate, and apoptosis of hypertrophic chondrocytes [78,79]. Estrogen receptors are both alpha and beta receptors. The alpha receptors are found in all zones of the growth plate, whereas the beta receptors are exclusively expressed in hypertrophic chondrocytes.

Obese children were found to display an increase in the level of androgens, and the excess of aromatase-containing tissues leads to a greater increase in systemic estrogen levels. This leads to accelerated growth, earlier puberty, premature epiphyseal fusion, and a shorter final adult height [80].

- Leptin

Leptin is a hormone secreted primarily by white adipose tissue. It regulates a variety of physiologic processes including food intake, body weight, immune function, bone growth, and remodeling. In the growth plates, Leptin was found to induce chondrocyte proliferation and differentiation by stimulating parathyroid hormone-related peptide (PTHrP) secretion and inhibiting Indian hedgehog homolog (Ihh) release [81]. In addition, it was found that leptin stimulates Hypoxia-Inducible Factor (HIF)-1, which is an important regulator of chondrocyte adaptation to their avascular and relatively hypoxic host tissues. HIF-1 is also important in the response of the growth plate to nutritional manipulation, playing a role in catch-up growth following a period of nutrient deprivation [82].

Obese children have a state of hypothalamic leptin resistance. While the brain may not be able to adequately respond to the increased leptin concentration, the growth plate does [80]. As leptin has receptors on the articular and physeal chondrocytes, elevated leptin has been shown to cause physeal pathology in animal models similar to the histologic changes seen at the physis affected by SCFE [83]. Leptin increases the width of the hypertrophic (maturation) and proliferative zones of the growth plate, leading to mechanical weakness associated with SCFE. This includes distortion and widening of the columnar architecture of the zone of hypertrophy and zone of proliferation [81,83]. Furthermore, increased leptin may lead to accelerated growth and epiphyseal growth plate closure [70].

#### 3.2.3. Major Paracrine Factors

- The Ihh-PTHrp Signaling Feedback Loop

Indian hedgehog homolog (Ihh) is one of the signaling proteins within the hedgehog signaling pathway and is regulated via PTHrP [84]. PTHrP binds and activates the PTH1R receptor in proliferating chondrocytes to stimulate their proliferation and delay their differentiation into pre-hypertrophic chondrocytes. Once there is sufficient proliferation and there are chondrocytes far enough from the PTHrP source, proliferation of these chondrocyte ends is termed pre-hypertrophic chondrocytes. These pre-hypertrophic chondrocytes have been found to synthesize Ihh, which will then signal back to the growth plate and lead to further PTHrP synthesis, directing the growing bone to continue proliferating and to grow in size. Thus, it can be stated that PTHrP is expressed at high levels in the periarticular resting cells of the growth plate, at lower levels in early proliferating chondrocytes, and once more at periarticular resting cartilage due to the action of Ihh from pre-hypertrophic chondrocytes. Thus, the Ihh/PTHrP signaling feedback loop regulates the pace of chondrocyte differentiation [6,85].

PTHrP is overexpressed in adipose tissue [84]. It has been found that overexpression of PTHrP on chondrocytes causes an excess of proliferation with delays in the proper ossification of bone. This delay can cause decreased bone strength and size [85].

- Fibroblast Growth Factors (FGFs)

The perichondrium of the postnatal growth plate expresses FGF 1, 2, 6, 7, 9, and 18. Expression of FGF 2, 7, 18, and 22 was observed in the plate itself [86]. Expression of their receptor proteins—FGFR1 and FGFR3—was also found. FGFR1 is expressed in both the perichondrium and hypertrophic chondrocytes, but not in proliferating chondrocytes. FGFR3 is expressed in the proliferating zone of the growth plate. FGFR1 and FGFR3 signaling are negative regulators of chondrocyte proliferation and differentiation [87,88,89]. In addition, FGF18 enhances vascular endothelial growth factor expression in hypertrophic chondrocytes, which indicates that FGF18 effects extend beyond the growth plate to integrate bone deposition and remodeling with growth plate activities [90]. Studies revealed that there is a link between Ihh/PTHrP signaling and the FGFR3 pathway in the epiphyseal growth plate. PTH1R activation attenuates chondrocyte expression of FGFR3,45, and FGFR3 signaling inhibits Ihh and PTH1R expression in the growth plate [91].

All the aforementioned FGFs are expressed in adipose tissue, and studies have shown that they may work in a paracrine manner to further cause adipogenesis [92]. Compounding the increased stress that obesity plays on developing bones and joints, the balance of FGF function in skeletal formation may also be altered.

- Wnt/β-Catenin Pathways

Wnts are signal transduction pathways that serve during development, after birth, and in the pathogenesis of many diseases [93]. Wnt signals are transduced by two distinct pathways: the canonical Wnt/β-catenin pathway and a β-catenin noncanonical pathway. The Wnt/β-catenin signaling is an important regulator of chondrocyte proliferation and differentiation. Wnt/β-catenin is expressed in the cytoplasm of proliferative and pre-hypertrophic chondrocytes to inhibit hypertrophy and endochondral ossification. In addition, its nuclear expression in the hypertrophic chondrocytes stimulates their terminal differentiation [94]. Through both expression patterns, the Wnt/β-catenin pathway serves a key role in the development of long bones.

- Vascular Endothelial Growth Factors (VEGFs)

VEGFs are angiogenic factors essential for embryonic development, bone formation, and tissue remodeling/healing [95,96,97]. VEGF is expressed in hypertrophic chondrocytes to stimulate angiogenesis for replacement of cartilage with trabecular bone during skeletal growth and regeneration. The absence of blood vessel invasion results in an increased hypertrophic zone and reduced hypertrophic chondrocyte apoptosis [97,98].

Obesity has been shown to elevate VEGF levels, as greater blood supply is required to deliver oxygen to the adipose tissue. In particular, higher quantities of visceral fat were found to be the greatest contributor to raising VEGF [99]. The role of osteoblastic cells in secreting VEGF aids in bone regeneration and healing processes, which allows for higher bone densities and a reduction in bone fracture risk [100].

### 3.3. Clinical Pathology Related to Improper Nutrition and Obesity

**Figure 2:**
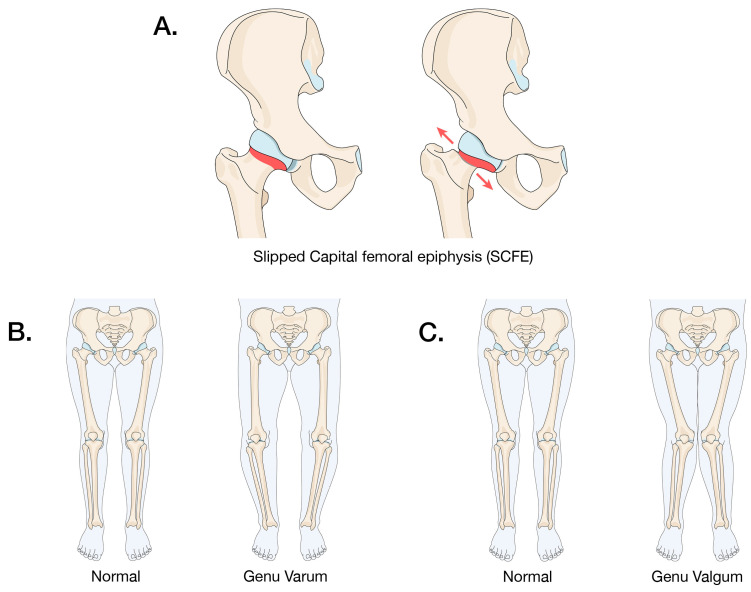
The possible clinical consequences of childhood obesity. The figure shows the possible clinical consequences of obesity in children. (**A**) Slipped capital femoral epiphysis in which the epiphysis of the femur separates from the metaphysis at the growth plate; (**B**) Genu Varum (bowed legs); and (**C**) Genu Valgum (knocked knees).

Obesity can act as a deforming force on the growing skeleton in genetically susceptible individuals. It is associated with a range of pediatric skeletal conditions (Figure 2) including Slipped Capital Femoral Epiphysis, Blount’s Disease (Genu Varum, or bowed legs) and Genu Valgum (knock knees).

#### 3.3.1. Slipped Capital Femoral Epiphysis (SCFE)

SCFE is a condition that affects the proximal femur growth plate in adolescents. Most patients with SCFE do not have an obvious endocrine abnormality, though the most common presenting abnormalities are hypothyroidism, growth hormone deficiency, and chronic renal failure. In patients without an endocrine abnormality, who constitute the vast majority of SCFE cases, mechanical and hormonal factors result in weakening of the proximal femoral physis, which leads to the slippage of the femoral epiphysis [70]. One potential association includes the altered leptin pathways and downstream effects seen in obese individuals and as evidenced in animal studies [80,81,83]. Such changes lead to altered growth patterns and ultimately weakening at the physis, thus potentially leading to SCFE.

There is a well-documented link between BMI and SCFE. In a study analyzing the BMI of SCFE patients compared to non-SCFE controls, the SCFE group had 81.1% of individuals with a BMI above the 95th percentile compared to 41.3% in the control group [101]. When compared to children of the same age, children with severe obesity had a 5.9 times higher risk of SCFE at ages 5 to 6, and a 17 times higher risk at ages 11 to 12 [102].

#### 3.3.2. Blount’s Disease (Genu Varum, or bowed legs)

Obesity and genetic factors are the main cause of pathologic Genu Varum. Infantile Genu Varum affects children from 2 to 5 years old, and adolescent Genu Varum affects children older than 10 years [103,104]. It is caused by abnormal compressive forces on the growth plate of the proximal tibia due to obesity. This leads to unequal physical growth with worsening severity being correlated with the degree of obesity [103]. This association between the magnitude of obesity and the degree of Genu Varum was found to be most pronounced with a patient under the age of 4 and with a BMI of more than 40 [104]. In a retrospective analysis of individuals with Blount’s Disease, the average BMI was 35.6, with early-onset patients having an average BMI of 29.2 and late-onset patients having an average BMI of 39.7 [103].

#### 3.3.3. Genu Valgum (Knocked Knees)

The normal physiological parameters of the hip–knee angle is around 12 degrees in healthy adults, and this normal alignment is usually achieved by the age of 7 [105]. Genu Valgum is typically described as a hip–knee angle greater than 15–20 degrees. In a study of the BMI of children with Genu Valgum, it was found that 71% of patients were overweight and 47% had a BMI of greater than 30 [106]. The severity of the deformity was directly associated with the degree of BMI and skeletal maturity, leading to increased compressive forces on the lateral knee growth plate. Older children were found to have a similar association between obesity and Genu Valgum. As the physiologic alignment of the knee after childhood is in minor Genu Valgum, the increase in compressive forces on the lateral side of the knee will exacerbate the Genu Valgum according to Hueter–Volkmann’s law [105,106].

## 4. Discussion

Most of the metabolic abnormalities related to childhood obesity are reversible with weight loss. For example, the GH abnormality in obese adolescents is completely reversible with normalization of the body weight [63]. There has been a reported decrease in the risk of bilateral SCFE in obese patients who lost weight [107]. Though the literature is sparse on the effects of losing weight for Genu Varum and Genu Valgum, one study did find that a one-pound decrease in weight resulted in a 4-fold reduction in the forces exerted on the knees [108]. Unfortunately, obese children are likely to grow into obese adults, and treatment programs designed for weight loss have a low success rate [106]. Thus, it is imperative to promote healthy lifestyle changes rather than focusing solely on a dieting approach to losing weight.

In reviewing normal physiology of the epiphyseal growth plate, it is a highly metabolic process dependent upon a complex network of nutrients, endocrine hormones, and local paracrine factors [9]. How these factors operate both independently and with one another to regulate the epiphyseal growth plate is dependent upon the parameters in which they are functioning, either under healthy weight or obese conditions. When comparing regulation under each of these parameters, it is evident that many of the necessary nutrients, hormones, and general processes are disrupted by effects resulting from an obese body habitus [14,15,16,17,22,52,55,56,63,73,74,80,92,99]. Thus, one must consider the potential for long-term skeletal abnormalities that may result from childhood obesity, and as such, practitioners should be proactive in treating patients with an increased BMI [2,10,18]. When approaching the treatment of childhood obesity, practitioners should focus on evidence-based nutritional guidelines, involve parental figures and community-based interventions, and should not focus solely on restrictive caloric diets to maintain optimal bone health [11,12,13]. The use of evidence-based nutritional guidelines can serve as a foundation for patients as they look for ways to improve dietary intake [11,12]. It may be beneficial to involve licensed nutritionists and/or dieticians to aid in the implementation and adherence of dietary recommendations. By involving parental figures and impactful community-based interventions, adherence can be further improved with the hopes of maximizing the positive effects of improved nutrition. Further, a change in dietary perspective from calorie restrictive to nutrient dense may aid both practitioners and patients alike to enable an enhanced sense of optimism and control to improve each patients’ disease state [11,16]. Last, when considering the multitude of factors influencing bone health, one must not neglect other valuable aspects of treatment, such as weight-bearing physical activity, that can help to enhance bone strength and improve the metabolic and hormonal profile of a patient seeking to counter the effects of childhood obesity [10,15,16].

Limitations include the evaluation of healthy weight and childhood obesity as steady states. Pediatric weight and body habitus is dynamically changing as one experiences various stages of growth and development during the early years and throughout puberty [5]. Thus, it may be valuable to consider how nutrient needs and hormone profiles change throughout dynamic stages of development.

As this study focused on the differences between healthy and obese parameters, future research may be warranted to investigate the physiological changes as one transitions from an obese condition to healthy weight. It may also be beneficial to evaluate best practices in treating obesity in pediatric patients of different ages as understanding of and adherence to treatment are likely to change along with development. Although there are areas for future research, we believe this study provides valuable evidence of what is required to maintain the epiphyseal growth plate, how it is affected by childhood obesity, and both why and how practitioners can treat childhood obesity to maintain the health of long bones in pediatric patients.

## Figures and Tables

**Table 1:** Nutritional summary for children based on age.

Current DRIs and RDAs	Ages (Years)	Male	Female
Calories (Kcal/day)	1–3	1000	1000
	4–8	1200	1400–1600
	9–13	1600	1800
	14–18	1800	2200–3200
Proteins (grams/day)	1–3	13	13
	4–8	19	19
	9–13	34	34
	14–18	46	52

**Table 2:** A summary of recommended daily pediatric mineral intake.

Current RDAs	Ages	Requirements
Calcium mg/day	0–6 months	200
	7–12 months	260
	1–3 years	700
	4–8 years	1000
	9–18 years	1300
Magnesium mg/day	0–6 months	30
	7–12 months	75
	1–3 years	80
	4–8 years	130
	9–13 years	240
	14–18 years	Male: 410 Female: 360
Fluoride mg/day	0–6 months	0.01
	7–12 months	0.5
	1–3 years	0.7
	4–8 years	1
	9–13 years	2
	14–18 years	3
Phosphate mg/day	0–6 months	100
	7–12 months	275
	1–3 years	460
	4–8 years	500
	9–18 years	1250
Zinc mg/day	0–6 months	2
	7–12 months	3
	1–3 years	3
	4–8 years	5
	9–13 years	8
	14–18 years	Male: 11 Female: 9
Iodine mcg/day	0–6 months	110
	7–12 months	130
	1–8 years	90
	9–13 years	120
	14–18 years	150

**Table 3:** A summary of recommended daily pediatric vitamin intake.

Current RAEs and RDAs	Ages	Requirement
Vitamin A mcg/day (RAE)	0–6 months	400
	7–12 months	500
	1–3 years	300
	4–8 years	400
	9–13 years	600
	14–18 years	900
Vitamin C mg/day	0–6 months	40
	7–12 months	50
	1–3 years	15
	4–8 years	25
	9–13 years	45
	14–18 years	M: 75 F: 65
Vitamin D IU/day	0–12 months	400
	1–18 years	600

## References

[1] Price C.T., Langford J.R., Liporace F.A. (2012). Essential Nutrients for Bone Health and a Review of their Availability in the Average North American Diet. Open Orthop. J..

[2] Ogden C.L., Fryar C.D., Martin C.B., Freedman D.S., Carroll M.D., Gu Q., Hales C.M. (2021). Trends in obesity prevalence by race and hispanic origin—1999–2000 to 2017–2018. JAMA J. Am. Med. Assoc..

[3] Seeman E., Delmas P.D. (2006). Bone Quality—The Material and Structural Basis of Bone Strength and Fragility. N. Engl. J. Med..

[4] Abad V., Meyers J.L., Weise M., Gafni R.I., Barnes K.M., Nilsson O.L.A., Bacher J.D., Baron J. (2002). The role of the resting zone in growth plate chondrogenesis. Endocrinology.

[5] Tanner J.M., Davies P.S.W. (1985). Clinical longitudinal standards for height and height velocity for North American children. J. Pediatr..

[6] Kronenberg H.M. (2003). Developmental regulation of the growth plate. Nature.

[7] Farnum C.E., Wilsman N.J. (1987). Morphologic stages of the terminal hypertrophic chondrocyte of growth plate cartilage. Anat. Rec..

[8] Burdan F., Szumiło J., Korobowicz A., Farooquee R., Patel S., Patel A., Patel A., Dave A., Szumiło M., Solecki M. (2009). Morphology and physiology of the epiphyseal growth plate. Folia Histochem. Cytobiol..

[9] Florencio-Silva R., da Sasso G.R.S., Sasso-Cerri E., Simões M.J., Cerri P.S. (2015). Biology of Bone Tissue: Structure, Function, and Factors That Influence Bone Cells. Biomed. Res. Int..

[10] Gutin B., Stallmann-Jorgensen I., Le A., Johnson M., Dong Y. (2011). Relations of Diet and Physical Activity to Bone Mass and Height in Black and White Adolescents. Pediatr Rep..

[11] Greer F.R., Krebs N.F., Baker R.D. (2006). Optimizing bone health and calcium intakes of infants, children, and adolescents. Pediatrics.

[12] Institute of Medicine (2006). Dietary Reference Intakes: The Essential Guide to Nutrient Requirements.

[13] Lui J.C. (2017). Nutritional Regulation of the Growth Plate. The Biology of the First 1000 Days.

[14] Upadhyay J., Farr O.M., Mantzoros C.S. (2015). The role of leptin in regulating bone metabolism. Metabolism.

[15] Soliman A., De Sanctis V., Elalaily R., Bedair S. (2014). Advances in pubertal growth and factors influencing it: Can we increase pubertal growth?. Indian J. Endocrinol. Metab..

[16] Soliman A., De Sanctis V., Elalaily R. (2014). Nutrition and pubertal development. Indian J. Endocrinol. Metab..

[17] Soliman A.T., Hassan A.E.H.I., Aref M.K., Hintz R.L., Rosenfeld R.G., Rogol A.D. (1986). Serum Insulin-Like Growth Factors I and II Concentrations and Growth Hormone and Insulin Responses to Arginine Infusion in Children with Protein-Energy Malnutrition before and after Nutritional Rehabilitation. Pediatr. Res..

[18] Millward D.J. (2017). Nutrition, infection and stunting: The roles of deficiencies of individual nutrients and foods, and of inflammation, as determinants of reduced linear growth of children. Nutr. Res. Rev..

[19] Gat-Yablonski G., Yackobovitch-Gavan M., Phillip M. (2017). Which dietary components modulate longitudinal growth?. Curr. Opin. Clin. Nutr. Metab. Care.

[20] Van Vught A.J., Heitmann B.L., Nieuwenhuizen A.G., Veldhorst M.A., Andersen L.B., Hasselstrom H., Brummer R.-J.M., Westerterp-Plantenga M.S. (2010). Association between intake of dietary protein and 3-year-change in body growth among normal and overweight 6-year-old boys and girls (CoSCIS). Public Health Nutr..

[21] Villamor E., Jansen E.C. (2016). Nutritional Determinants of the Timing of Puberty. Annu. Rev. Public Health.

[22] Wallach S. (1990). Effects of magnesium on skeletal metabolism. Magnes Trace Elem..

[23] Zimmermann M.B. (2011). The role of iodine in human growth and development. Semin. Cell Dev. Biol..

[24] Vannucci L., Fossi C., Quattrini S., Guasti L., Pampaloni B., Gronchi G., Giusti F., Romagnoli C., Cianferotti L., Marcucci G. (2018). Calcium Intake in Bone Health: A Focus on Calcium-Rich Mineral Waters. Nutrients..

[25] Murray E.J.B., Murray S.S., Grisanti M., Duarte M.E.L., Urist M.R. (1997). Effect of low dietary calcium on bone metabolism in the SENCAR mouse. J. Orthop. Res..

[26] Schrager S. (2005). Dietary Calcium Intake and Obesity. J. Am. Board Fam. Med..

[27] Abrams S.A., Chen Z., Hawthorne K.M. (2014). Magnesium metabolism in 4-year-old to 8-year-old children. J. Bone Min. Res..

[28] Lau K.H.W., Baylink D.J. (1998). Molecular mechanism of action of fluoride on bone cells. J. Bone Min. Res..

[29] Piuri G., Zocchi M., Della Porta M., Ficara V., Manoni M., Zuccotti G.V., Pinotti L., Maier J.A., Cazzola R. (2021). Magnesium in Obesity, Metabolic Syndrome, and Type 2 Diabetes. Nutrients.

[30] Rude R.K., Gruber H.E., Wei L.Y., Frausto A., Mills B.G. (2003). Magnesium Deficiency: Effect on Bone and Mineral Metabolism in the Mouse. Calcif. Tissue Int..

[31] Whitford G.M. (1999). Fluoride metabolism and excretion in children. J. Public Health Dent..

[32] Penido M.G.M.G., Alon U.S. (2012). Phosphate homeostasis and its role in bone health. Pediatr. Nephrol..

[33] Yesİldag A., Heybeli N., Candır O., Oyar O., Baykal B., Mumcu E.F., Gulsoy U.K. (2004). Effects of fluoride on growth plate cartilage in rats: Radiological and histopathological findings. Flouride.

[34] Gao Y., Gui F., Li D., Zhang R., Sun Q., Guo X. (2020). Fluoride regulates the expression of extracellular matrix HSPG and related signaling pathways FGFR3 and Ihh/PTHrP feedback loop during endochondral ossification. Env. Toxicol. Pharmacol..

[35] Liu L., Wang M., Li Y., Liu H., Hou C., Zeng Q., Li P., Zhao Q., Dong L., Yu X. (2019). Low-to-moderate fluoride exposure in relation to overweight and obesity among school-age children in China. Ecotoxicol. Environ. Saf..

[36] MacDonald R.S. (2000). The Role of Zinc in Growth and Cell Proliferation. J. Nutr..

[37] Al Jurayyan N.A.M., Mohamed S., Al Issa S.D.A., Al Jurayyan A.N.A. (2012). Rickets and osteomalacia in Saudi children and adolescents attending endocrine clinic, Riyadh, Saudi Arabia. Sudan J. Paediatr..

[38] Golden N.H., Abrams S.A., Daniels S.R., Abrams S.A., Corkins M.R., de Ferranti S.D., Golden N.H., Magge S.N., Schwarzenberg S.J. (2014). Optimizing Bone Health in Children and Adolescents. Pediatrics.

[39] Bosman A., Campos-Obando N., Medina-Gomez C., Voortman T., Uitterlinden A.G., Zillikens M.C. (2022). Serum Phosphate, BMI, and Body Composition of Middle-Aged and Older Adults: A Cross-Sectional Association Analysis and Bidirectional Mendelian Randomization Study. J. Nutr..

[40] Huang T., Yan G., Guan M. (2020). Zinc Homeostasis in Bone: Zinc Transporters and Bone Diseases. Int. J. Mol. Sci..

[41] Prasad A.S. (2004). Zinc deficiency: Its characterization and treatment. Met. Ions Biol. Syst..

[42] Institute of Medicine (2001). Dietary Reference Intakes for Vitamin A, Vitamin K, Arsenic, Boron, Chromium, Copper, Iodine, Iron, Manganese, Molybdenum, Nickel, Silicon, Vanadium, and Zinc.

[43] Rossi L., Migliaccio S., Corsi A., Marzia M., Bianco P., Teti A., Gambelli L., Cianfarani S., Paoletti F., Branca F. (2001). Reduced Growth and Skeletal Changes in Zinc-Deficient Growing Rats Are Due to Impaired Growth Plate Activity and Inanition. J. Nutr..

[44] Kurtogu S., Patiroglu T.E., Karakas S.E. (1987). Effect of growth hormone on epiphyseal growth plates in zinc deficiency. Tokai J. Exp. Clin. Med..

[45] Khorsandi H., Nikpayam O., Yousefi R., Parandoosh M., Hosseinzadeh N., Saidpour A., Ghorbani A. (2019). Zinc supplementation improves body weight management, inflammatory biomarkers and insulin resistance in individuals with obesity: A randomized, placebo-controlled, double-blind trial. Diabetol. Metab. Syndr..

[46] Liu M.J., Bao S., Bolin E.R., Burris D.L., Xu X., Sun Q., Killilea D.W., Shen Q., Ziouzenkova O., Belury M.A. (2013). Zinc Deficiency Augments Leptin Production and Exacerbates Macrophage Infiltration into Adipose Tissue in Mice Fed a High-Fat Diet1–3. J. Nutr..

[47] Shaikh M.A., Naeem Z., Alshahat A.A. (2013). Growth Plate Changes Associated with Hypothyroidism amongst the Pre and Postnatal Rats. Int. J. Health Sci..

[48] Herlihy J.T., Stacy C., Bertrand H.A. (1990). Long-term food restriction depresses serum thyroid hormone concentrations in the rat. Mech. Ageing Dev..

[49] Moleti M., Di Mauro M., Paola G., Olivieri A., Vermiglio F. (2021). Nutritional iodine status and obesity. Thyroid. Res..

[50] Conaway H.H., Henning P., Lerner U.H. (2013). Vitamin A Metabolism, Action, and Role in Skeletal Homeostasis. Endocr. Rev..

[51] De Luca F., Uyeda J.A., Mericq V., Mancilla E.E., Yanovski J.A., Barnes K.M., Zile M.H., Baron J. (2000). Retinoic Acid Is a Potent Regulator of Growth Plate Chondrogenesis. Endocrinology.

[52] García O.P. (2012). Effect of vitamin A deficiency on the immune response in obesity. Proc. Nutr. Soc..

[53] Aghajanian P., Hall S., Wongworawat M.D., Mohan S. (2015). The Roles and Mechanisms of Actions of Vitamin C in Bone: New Developments. J. Bone Miner. Res..

[54] Liakakos D., Vlachos P., Doulas N.L., Litsios B., Alexiou D. (1981). Effect of Ascorbic Acid (Vitamin C) on the Epiphyseal Plate of Young Guinea Pigs Receiving Prednisolone. Dev. Pharmacol. Ther..

[55] Goltzman D. (2018). Functions of vitamin D in bone. Histochem. Cell Biol..

[56] Carr A.C., Rowe S. (2020). Factors Affecting Vitamin C Status and Prevalence of Deficiency: A Global Health Perspective. Nutrients.

[57] Khazai N., Judd S.E., Tangpricha V. (2008). Calcium and vitamin D: Skeletal and extraskeletal health. Curr. Rheumatol. Rep..

[58] Lin R., Amizuka N., Sasaki T., Aarts M.M., Ozawa H., Goltzman D., Henderson J.E., White J.H. (2002). 1α,25-Dihydroxyvitamin D3 Promotes Vascularization of the Chondro-osseous Junction by Stimulating Expression of Vascular Endothelial Growth Factor and Matrix Metalloproteinase 9. J. Bone Miner. Res..

[59] Ballesteros M., Leung K.-C., Ross R.J.M., Iismaa T.P., Ho K.K.Y. (2000). Distribution and Abundance of Messenger Ribonucleic Acid for Growth Hormone Receptor Isoforms in Human Tissues1. J. Clin. Endocrinol. Metab..

[60] Dupont J., Holzenberger M. (2003). Biology of insulin-like growth factors in development. Birth Defects Res. C Embryo Today.

[61] Mehls O., Himmele R., Hömme M., Kiepe D., Klaus G. (2001). The interaction of glucocorticoids with the growth hormone-insulin-like growth factor axis and its effects on growth plate chondrocytes and bone cells. J. Pediatr. Endocrinol. Metab..

[62] Siebler T., Robson H., Shalet S.M., Williams G.R. (2001). Glucocorticoids, Thyroid Hormone and Growth Hormone Interactions: Implications for the Growth Plate. Horm. Res. Paediatr..

[63] Scacchi M., Pincelli A., Cavagnini F. (1999). Growth hormone in obesity. Int. J. Obes..

[64] Kopp P. (2001). Human Genome and Diseases: Review—The TSH receptor and its role in thyroid disease. Cell. Mol. Life Sci..

[65] Ballock R.T., Reddi A.H. (1994). Thyroxine is the serum factor that regulates morphogenesis of columnar cartilage from isolated chondrocytes in chemically defined medium. J. Cell Biol..

[66] Ballock R.T., Zhou X., Mink L.M., Chen D.H.C., Mita B.C., Stewart M.C. (2000). Expression of Cyclin-Dependent Kinase Inhibitors in Epiphyseal Chondrocytes Induced to Terminally Differentiate with Thyroid Hormone. Endocrinology.

[67] Ishikawa Y., Genge B.R., Wuthier R.E., Wu L.N.Y. (1998). Thyroid Hormone Inhibits Growth and Stimulates Terminal Differentiation of Epiphyseal Growth Plate Chondrocytes. J. Bone Miner. Res..

[68] Moez Ali B.A., Mahrous D.M. (2016). Thyroid Function Status in Obese Children. J. Diabetes Metab..

[69] Reinehr T. (2002). Thyroid hormones before and after weight loss in obesity. Arch. Dis. Child.

[70] Witbreuk M., van Kemenade F.J., van der Sluijs J.A., Jansma E.P., Rotteveel J., van Royen B.J. (2013). Slipped capital femoral epiphysis and its association with endocrine, metabolic and chronic diseases: A systematic review of the literature. J. Child Orthop..

[71] Van der Eerden B.C.J., Karperien M., Wit J.M. (2003). Systemic and Local Regulation of the Growth Plate. Endocr. Rev..

[72] Ahmed S.F., Sävendahl L. (2009). Promoting Growth in Chronic Inflammatory Disease: Lessons from Studies of the Growth Plate. Horm. Res. Paediatr..

[73] Chu L., Sheng K., Liu P., Ye K., Wang Y., Li C., Kang X. (2017). Increased Cortisol and Cortisone Levels in Overweight Children. Med. Sci. Monit. Basic Res..

[74] Veldhorst M.A., Noppe G., Jongejan M.H., Kok C.B., Mekic S., Koper J.W., van Rossum E.F., van den Akker E.L. (2014). Increased Scalp Hair Cortisol Concentrations in Obese Children. J. Clin. Endocrinol. Metab..

[75] Wongdee K., Krishnamra N., Charoenphandhu N. (2012). Endochondral bone growth, bone calcium accretion, and bone mineral density: How are they related?. J. Physiol. Sci..

[76] Keenan B.S., Richards G.E., Ponder S.W., Dallas J.S., Nagamani M., Smith E.R. (1993). Androgen-stimulated pubertal growth: The effects of testosterone and dihydrotestosterone on growth hormone and insulin-like growth factor-I in the treatment of short stature and delayed puberty. J. Clin. Endocrinol. Metab..

[77] Oz O., Millsaps R., Welch R., Birch J., Zerwekh J. (2001). Expression of aromatase in the human growth plate. J. Mol. Endocrinol..

[78] Norjavaara E., Ankarberg C., Albertsson-Wikland K. (1996). Diurnal rhythm of 17 beta-estradiol secretion throughout pubertal development in healthy girls: Evaluation by a sensitive radioimmunoassay. J. Clin. Endocrinol. Metab..

[79] Juul A. (2001). The effects of oestrogens on linear bone growth. Hum. Reprod. Update.

[80] Shalitin S., Kiess W. (2017). Putative Effects of Obesity on Linear Growth and Puberty. Horm. Res. Paediatr..

[81] Gat-Yablonski G., Shtaif B., Phillip M. (2007). Leptin Stimulates Parathyroid Hormone Related Peptide Expression in the Endochondral Growth Plate. J. Pediatr. Endocrinol. Metab..

[82] Even-Zohar N., Jacob J., Amariglio N., Rechavi G., Potievsky O., Phillip M., Gat-Yablonski G. (2008). Nutrition-induced catch-up growth increases hypoxia inducible factor 1α RNA levels in the growth plate. Bone.

[83] Halverson S.J., Warhoover T., Mencio G.A., Lovejoy S.A., Martus J.E., Schoenecker J.G. (2017). Leptin Elevation as a Risk Factor for Slipped Capital Femoral Epiphysis Independent of Obesity Status. J. Bone Jt. Surgery.

[84] Izquierdo-Lahuerta A. (2021). The Parathyroid Hormone-Related Protein/Parathyroid Hormone 1 Receptor Axis in Adipose Tissue. Biomolecules.

[85] Hallett S.A., Ono W., Ono N. (2019). Growth Plate Chondrocytes: Skeletal Development, Growth and Beyond. Int. J. Mol. Sci..

[86] Lazarus J.E., Hegde A., Andrade A.C., Nilsson O., Baron J. (2007). Fibroblast growth factor expression in the postnatal growth plate. Bone.

[87] Jacob A.L., Smith C., Partanen J., Ornitz D.M. (2006). Fibroblast growth factor receptor 1 signaling in the osteo-chondrogenic cell lineage regulates sequential steps of osteoblast maturation. Dev. Biol..

[88] Deng C., Wynshaw-Boris A., Zhou F., Kuo A., Leder P. (1996). Fibroblast Growth Factor Receptor 3 Is a Negative Regulator of Bone Growth. Cell.

[89] Peters K., Ornitz D., Werner S., Williams L. (1993). Unique Expression Pattern of the FGF Receptor 3 Gene during Mouse Organogenesis. Dev. Biol..

[90] Liu Z., Lavine K.J., Hung I.H., Ornitz D.M. (2007). FGF18 is required for early chondrocyte proliferation, hypertrophy and vascular invasion of the growth plate. Dev. Biol..

[91] Chen L. (2001). A Ser365→Cys mutation of fibroblast growth factor receptor 3 in mouse downregulates Ihh/PTHrP signals and causes severe achondroplasia. Hum. Mol. Genet..

[92] Gabrielsson B.G., Johansson J.M., Jennische E., Jernås M., Itoh Y., Peltonen M., Olbers T., Lönn L., Lönroth H., Sjöström L. (2002). Depot-Specific Expression of Fibroblast Growth Factors in Human Adipose Tissue. Obes. Res..

[93] Huang H., He X. (2008). Wnt/β-catenin signaling: New (and old) players and new insights. Curr. Opin. Cell Biol..

[94] Tamamura Y., Otani T., Kanatani N., Koyama E., Kitagaki J., Komori T., Yamada Y., Costantini F., Wakisaka S., Pacifici M. (2005). Developmental Regulation of Wnt/β-Catenin Signals Is Required for Growth Plate Assembly, Cartilage Integrity, and Endochondral Ossification. J. Biol. Chem..

[95] Ferrara N., Carver-Moore K., Chen H., Dowd M., Lu L., O’Shea K.S., Powell-Braxton L., Hillan K.J., Moore M.W. (1996). Heterozygous embryonic lethality induced by targeted inactivation of the VEGF gene. Nature.

[96] Street J., Bao M., de Guzman L., Bunting S., Peale F.V., Ferrara N., Steinmetz H., Hoeffel J., Cleland J.L., Daugherty A. (2002). Vascular endothelial growth factor stimulates bone repair by promoting angiogenesis and bone turnover. Proc. Natl. Acad. Sci. USA.

[97] Horner A., Bord S., Kelsall A.W., Coleman N., Compston J.E. (2001). Tie2 ligands angiopoietin-1 and angiopoietin-2 are coexpressed with vascular endothelial cell growth factor in growing human bone. Bone.

[98] Haigh J.J., Gerber H.-P., Ferrara N., Wagner E.F. (2000). Conditional inactivation of VEGF-A in areas of collagen2a1 expression results in embryonic lethality in the heterozygous state. Development.

[99] Zaki M.E., Basha W., Yousef R.N., Awad M. (2019). Serum Vascular Endothelial Growth Factor in Egyptian Obese Women with Insulin Resistance. Open Access Maced. J. Med. Sci..

[100] Hu K., Olsen B.R. (2016). The roles of vascular endothelial growth factor in bone repair and regeneration. Bone.

[101] Manoff E.M., Banffy M.B., Winell J.J. (2005). Relationship Between Body Mass Index and Slipped Capital Femoral Epiphysis. J. Pediatr. Orthop..

[102] Perry D.C., Metcalfe D., Lane S., Turner S. (2018). Childhood Obesity and Slipped Capital Femoral Epiphysis. Pediatrics.

[103] Kgoedi M., Rischbieter P., Goller R. (2019). Body mass index and Blount’s disease: A single academic hospital experience. SA Orthop. J..

[104] Sabharwal S., Zhao C., McClemens E. (2007). Correlation of Body Mass Index and Radiographic Deformities in Children with Blount Disease. J. Bone Jt. Surg..

[105] Blasier R.D. (2008). Tachdjian’s Pediatric Orthopaedics, 4th Edition. J. Bone Jt. Surg..

[106] Walker J.L., Hosseinzadeh P., White H., Murr K., Milbrandt T.A., Talwalkar V.J., Iwinski H., Muchow R. (2019). Idiopathic Genu Valgum and Its Association with Obesity in Children and Adolescents. J. Pediatr. Orthop..

[107] Nasreddine A.Y., Heyworth B.E., Zurakowski D., Kocher M.S. (2013). A Reduction in Body Mass Index Lowers Risk for Bilateral Slipped Capital Femoral Epiphysis. Clin. Orthop. Relat. Res..

[108] Messier S.P., Resnik A.E., Beavers D.P., Mihalko S.L., Miller G.D., Nicklas B.J., DeVita P., Hunter D.J., Lyles M.F., Eckstein F. (2018). Intentional Weight Loss in Overweight and Obese Patients with Knee Osteoarthritis: Is More Better?. Arthritis Care Res..

